# Intrinsic disorder in Viral Proteins Genome-Linked: experimental and predictive analyses

**DOI:** 10.1186/1743-422X-6-23

**Published:** 2009-02-16

**Authors:** Eugénie Hébrard, Yannick Bessin, Thierry Michon, Sonia Longhi, Vladimir N Uversky, François Delalande, Alain Van Dorsselaer, Pedro Romero, Jocelyne Walter, Nathalie Declerk, Denis Fargette

**Affiliations:** 1 UMR 1097 Résistance des Plantes aux Bio-agresseurs, IRD, CIRAD, Université de Montpellier II, BP 64501, 34394 Montpellier cedex 5, France; 2 Centre de Biochimie Structurale, UMR 5048, 29 rue de Navacelles, 34090 Montpellier, France; 3 UMR1090 Génomique Diversité Pouvoir Pathogène, INRA, Université de Bordeaux 2, F-33883 Villenave D'Ornon, France; 4 UMR 6098 Architecture et Fonction des Macromolécules Biologiques, CNRS, Universités Aix-Marseille I et II, Campus de Luminy, 13288 Marseille Cedex 09, France; 5 Center for Computational Biology and Bioinformatics, Department of Biochemistry and Molecular Biology, Indiana University School of Medicine, Indianapolis, IN 46202, USA; 6 Institute for Biological Instrumentation, Russian Academy of Sciences, 142290 Pushchino, Moscow Region, Russia; 7 Laboratoire de Spectrométrie de Masse Bio-Organique, ECPM, 67087 Strasbourg, France

## Abstract

**Background:**

VPgs are viral proteins linked to the 5' end of some viral genomes. Interactions between several VPgs and eukaryotic translation initiation factors eIF4Es are critical for plant infection. However, VPgs are not restricted to phytoviruses, being also involved in genome replication and protein translation of several animal viruses. To date, structural data are still limited to small picornaviral VPgs. Recently three phytoviral VPgs were shown to be natively unfolded proteins.

**Results:**

In this paper, we report the bacterial expression, purification and biochemical characterization of two phytoviral VPgs, namely the VPgs of *Rice yellow mottle virus *(RYMV, genus *Sobemovirus*) and *Lettuce mosaic virus *(LMV, genus *Potyvirus*). Using far-UV circular dichroism and size exclusion chromatography, we show that RYMV and LMV VPgs are predominantly or partly unstructured in solution, respectively. Using several disorder predictors, we show that both proteins are predicted to possess disordered regions. We next extend theses results to 14 VPgs representative of the viral diversity. Disordered regions were predicted in all VPg sequences whatever the genus and the family.

**Conclusion:**

Based on these results, we propose that intrinsic disorder is a common feature of VPgs. The functional role of intrinsic disorder is discussed in light of the biological roles of VPgs.

## Background

The interactions between eukaryotic translation initiation factors eIF4Es and Viral proteins genome-linked (VPgs) are critical for plant infection by potyviruses (for review see [1]). Mutations in plant eIF4Es result in recessive resistances [2-7]. Mutations in VPgs of several potyviruses result in resistance-breaking isolates [7-14]. These interactions were demonstrated *in vitro *by interaction assays and *in planta *by mean of co-localisation experiments [15-22]. Their exact roles are still unclear, although VPg/eIF4E interactions had been suggested to be involved in protein translation, in RNA replication and in cell-to-cell movement (for review see [23]). A similar interaction has been postulated in the rice/*Rice yellow mottle virus *(RYMV, *Sobemovirus*) pathosystem, involving the virulence factor VPg and the resistance factor eIF(iso)4G [24].

Recently, *Sesbania mosaic virus *(SeMV, genus *Sobemovirus*), *Potato virus Y *(PVY, genus *Potyvirus*) and *Potato virus A *(PVA, genus *Potyvirus*) VPgs were reported to be "natively unfolded proteins" [25-27]. Natively unfolded proteins, also called intrinsically disordered proteins (IDPs), lack a unique 3D-structure and exist as a dynamic ensemble of conformations at physiological conditions. Proteins may be partially or fully intrinsically disordered, possessing a wide range of conformations depending on the degree of disorder. Disordered domains have been grouped into at least two broad classes – compact (molten globule-like) and extended (natively unfolded proteins) [28,29]. IDPs possess a number of crucial biological functions including molecular recognition and regulation [30-37]. The functional diversity provided by disordered regions is believed to complement functions of ordered protein regions by protein-protein interactions [38-40].

Intrinsically unstructured proteins and regions differ from structured globular proteins and domains with regard to many attributes, including amino acid composition, sequence complexity, hydrophobicity, charge, flexibility, and type and rate of amino acid substitutions over evolutionary time. Many of these differences were utilized to develop various algorithms for predicting intrinsic order and disorder from amino acid sequences [41,42]. Bioinformatic analyses using disorder predictors showed that a surprisingly high percentage of genome putative coding sequences are intrinsically disordered. Eukaryotes genomes would encode more disordered proteins than prokaryotes having 52–67% of their translated products containing segments predicted to have more than 40 consecutive disordered residues [43-47]. The highest proportion of conserved predicted disordered regions (PDRs) is found in protein domains involved in protein-protein transient interactions (signalling and regulation). So far, disorder prediction data for viral proteins are scarce, although viruses have been shown to contain the highest proportion of proteins containing conserved predicted disordered regions (PDRs) compared to archaea, bacteria and eukaryota [48].

The presence of VPgs is not restricted to poty- and sobemoviruses but is also found in animal viruses with double or positive single strand (ss) RNA genome belonging to several unrelated virus families and genera. The term "VPg" refers to proteins highly diverse in sequence and in size (2–4 kDa for *Picornaviridae *and *Comoviridae *members, 10–26 kDa for *Potyviridae*, *Sobemoviruses *and *Caliciviridae *members, and up to 90 kDa for *Birnaviridae *members) [23]. High-resolution structural data are limited to 2–4 kDa VPgs. The 3D structures of synthetic peptides corresponding to *Picornaviridae *VPgs are the only ones available to date [49-51].

In this paper, we report the bacterial expression, purification and biochemical characterization of VPgs from *Rice yellow mottle virus *(RYMV) and *Lettuce mosaic virus *(LMV), two viruses of agronomic interest related to SeMV (genus *Sobemovirus*) or PVY and PVA (genus *Potyvirus*). We show that they both contain disordered regions although at a different extent. We next extend these results to a set of 14 VPg sequences representative of the various viral species. In particular, we focused on viruses for which functional VPg domains have been mapped, and in particular to those viruses the VPgs of which are known to interact with translation initiation factors. The disorder propensities of the 14 VPg sequences were assessed *in silico *using several complementary disorder predictors. Finally, the possible implications of structural disorder of VPgs in light of to their biological functions are discussed.

## Results

### Experimental evidences of intrinsic disorder in RYMV and LMV VPgs

**Figure 1:**
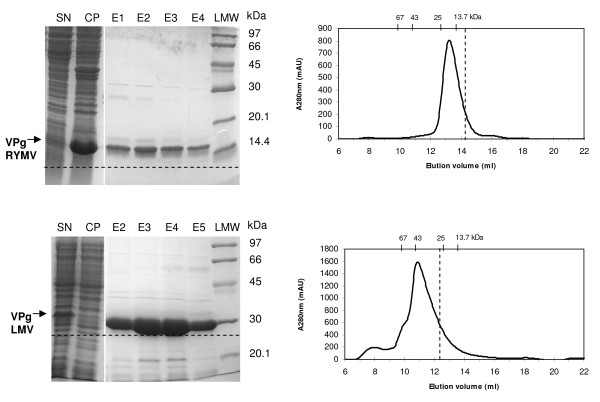
**Electrophoretic mobility and size-exclusion chromatography profile of RYMV and LMV VPgs**. A, C. 15% SDS-PAGE of recombinant His-tagged RYMV and LMV VPgs recovered from the supernatant (SN) and from the cell pellet (CP) after *E. coli *cell extraction, and after imidazole gradient elution fractions (E1 to E5) obtained after loading a 1 ml affinity nickel column (GE Healthcare) with the soluble fraction of the bacterial lysate. Low molecular weight (LMW) protein standards for SDS PAGE (GE Healthcare) are shown. The expected molecular masses of 10.53 and 26.25 kDa respectively were indicated by broken lines. The proteins in the major band (indicated by an arrow) migrate with an apparent molecular mass of about 15 and 27 kDa, respectively. B, D. Elution profile of purified His-tagged VPgs from a Superdex 75 HR10/30 column (GE Healthcare) in 50 mM Tris-HCl pH 8, 300 mM NaCl, at a flow rate of 0.5 ml/min. The proteins were eluted in a major peak with an apparent molecular mass of about 17 and 40 kDa respectively as deduced from column calibration with low molecular weight protein standards for gel filtration (GE Healthcare).

In order to assess the possible disordered state of RYMV and LMV VPgs, two members of the sobemo- and potyviruses respectively, we undertook their bacterial expression, purification and biochemical characterization. For this purpose, both proteins were produced as His-tagged fusion in *E. coli*. By contrast to LMV VPg, most of the recombinant RYMV VPg was produced as inclusion bodies and only a small fraction could be recovered from the cell extract supernatant under native conditions (Figure 1A and 1C). Mass spectrometry confirmed that purified RYMV and LMV VPgs have the expected molecular masses, 10.53 and 26.25 kDa respectively. However, their apparent molecular masses turned out to be higher as judged by SDS-PAGE and/or size exclusion chromatography (Figure 1). RYMV VPg migrated at around 15 kDa in denaturating conditions whereas no such discrepancy was observed in the case of LMV VPg (Figure 1A and 1C). Abnormal mobility in denaturating electrophoresis has been already previously described for IDPs (see [52] and references therein cited) and is due to their high proportion of acidic residues (25% for RYMV VPg compared to 15% for LMV VPg) [33]. Upon gel filtration, both RYMV and LMV VPgs showed apparent larger molecular masses of 17 and 40 kDa respectively. Natively unfolded proteins have an increased hydrodynamic volume compared to globular proteins (see [52] and references therein cited). The electrophoretic and hydrodynamic behaviors of RYMV and LMV VPgs suggest that these proteins are not folded as globular proteins.

**Figure 2:**
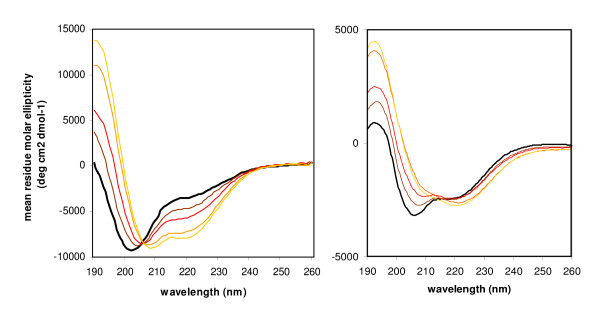
**Far UV-CD spectra of RYMV and LMV VPgs**. CD spectra of purified RYMV (A) and LMV VPgs (B) in the absence (black line) or in the presence of 5% (brown line), 10% (red line), 20% (orange line) and 30% (yellow line) of TFE.

The structural properties of the recombinant VPgs were investigated by far UV-circular dichroism (far-UV CD). The CD spectrum of the RYMV VPg purified in non-denaturating conditions is typical of an intrinsically disordered protein, as judged from its large negative ellipticity near 200 nm and from its low ellipticity at 190 nm (Figure 2A). As reported by Uversky et al., far-UV CD enables discrimination between random coils and pre-molten globules, based on the ratio of the ellipticity values at 200 and 222 nm [28]. In the case of RYMV VPg, the ellipticity values of -8830 and -3324 degrees cm^2 ^dmol^-1 ^at 200 and 222 nm respectively are consistent with the existence of some residual secondary structure, characteristic of the pre-molten globule state. The disordered state of LMV VPg is much less pronounced (Figure 2B): indeed, the CD spectrum is indicative of a predominantly folded protein, as judged based on the presence of two well-defined minima at 208 and 222 nm and by the positive ellipticity at 190 nm. Nevertheless, the relatively low ellipticity at 190 nm and the slightly negative ellipticity near 200 nm of 621 and -1573 degrees cm^2 ^dmol^-1 ^respectively, are indicative of the presence of disordered regions (Figure 2B).

Previous secondary structure predictions have suggested that both RYMV and LMV VPgs contain a high proportion of α-helices, 35% and 33% respectively [21,24]. The secondary structure stabilizer 2,2,2-trifluoroethanol (TFE) was therefore used to test the propensity of these proteins to undergo induced folding into an α-helical conformation. The gain of α-helicity by both VPgs, as judged based on the characteristic maximum at 190 nm and minima at 208 and 222 nm, parallels the increase in TFE concentration (Figure 2). The α-helical propensity of VPgs is revealed at TFE concentrations as low as 5%. Further calculations carried out with the K2d program [53] indicated an α-helix content of 30% (± 4%) for RYMV VPg in the presence of 30% TFE.

### Disorder predictions in sobemoviral VPgs

**Figure 3:**
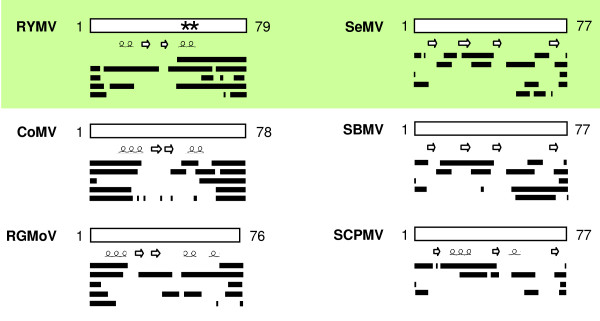
**Disorder predictions of sobemoviral VPgs**. Five predictors were used: PONDR^® ^VLXT, FoldIndex^©^, DISOPRED2, VSL2, IUPred. The location of predicted disordered regions (in the order provided by the above-listed predictors) was schematically represented by lines along the VPg sequence. Numbering indicates the VPg length. The consensus predicted α-helices and β-strands are indicated. The sites involved in RYMV virulence (*) are indicated. The VPgs experimentally demonstrated to be disordered are shaded. RYMV *Rice yellow mottle virus*, CoMV *Cocksfoot mottle virus*, RGMoV *Ryegrass mottle virus*, SBMV *Southern bean mosaic virus*, SCPMV *Southern cowpea mosaic virus*, SeMV *Sesbania mottle virus*.

The disorder propensities of VPgs from six sobemoviruses including RYMV and SeMV were evaluated using five complementary per-residue predictors of intrinsic disorder (PONDR^® ^VLXT, FoldIndex^©^, DISOPRED2, PONDR^® ^VSL2 and IUPred). The amino acid sequences of sobemoviral VPgs are highly diverse (20% identity between RYMV and SeMV). Regions with a propensity to be disordered are predicted in all VPgs (Figure 3). The boundaries of PDRs varied depending on the virus and the prediction method. However, according to PDR distribution within the sequences, two groups of sobemoviral VPgs can be distinguished: RYMV/CoMV/RGMoV VPgs in one group and SeMV/SBMV/SCPMV VPgs in the other group. This classification is consistent with the phylogenetic relationships earlier described [54]. In the RYMV group, the N- and C-terminus of the protein are predicted to be disordered. The consensus secondary structure prediction in this group indicates the presence of an α-helix followed by two β-strands and another α-helix. Part of the terminal regions of these VPgs are predicted to have propensities both to be disordered and to be folded in α-helices. Residues 48 and 52, which are associated with RYMV virulence, are located in the C-terminal region [55]. These residues have been proposed to participate in the interaction with two antiparallel helices of the eIF(iso)4G central domain bearing E309 and E321, two residues involved in rice resistance [24]. In the second group, the consensus is more difficult to define and the PDRs are generally shorter. Three conserved β-strands are predicted in the members of this group. Despite the inconsistencies among predictors and the intra-species differences, a propensity to structural disorder is predicted in all sobemoviral VPgs including the SeMV VPg, which had been previously experimentally shown to be disordered [25].

### Disorder predictions in potyviral VPgs

**Figure 4:**
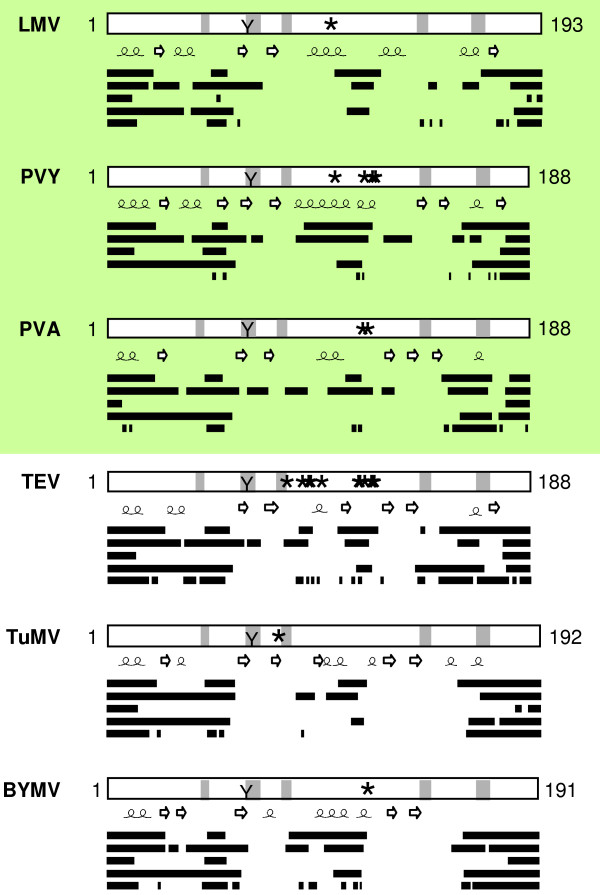
**Disorder predictions of potyviral VPgs**. Five predictors were used: PONDR^® ^VLXT, FoldIndex^©^, DISOPRED2, VSL2, IUPred. The location of predicted disordered (in the order provided by the above-listed predictors) was schematically represented by lines along the VPg sequence. Numbering indicates the VPg length. Highly conserved regions (grey) and consensus predicted α-helices and β-strands are indicated. The conserved tyrosine (Y) involved in VPg urydylylation and the sites (*) involved in virulence are indicated. The VPgs experimentally demonstrated to be disordered are shaded. LMV *Lettuce mosaic virus*, PVY *Potato virus Y*, PVA *Potato virus *A, TEV *Tobacco etch virus*, TuMV *Turnip mosaic virus*, BYMV *Bean yellow mosaic virus*.

The disorder propensity of six potyviral VPgs for which correlations between sequences and functions are well documented was evaluated. The sequence identity of these potyviruses ranges from 42% to 54%. Most of the highly conserved regions are within domains predicted to be ordered (Figure 4). However, PDRs were detected in each potyviral VPg, including PVY and PVA which have been shown to be intrinsically disordered [26,27]. The length of the disordered regions varies among potyviruses and discrepancies between results obtained with different predictors are observed. Nevertheless, the N- and C-terminal regions are predicted to be mainly disordered for all proteins (Figure 4). They contain two highly conserved segments spanning residues 43 to 45 and residues 165 to 170. Beyond the N- and C-terminus, the central region of the VPgs is also predicted to be disordered by some predictors. Several secondary structure elements are predicted along the proteins including the central putative disordered domain that is predicted to adopt an α-helical conformation. Interestingly, VPg sites involved in potyviral virulence are generally located in this internal PDR (Figure 4). This region fits perfectly with the domain of LMV VPg previously identified as a part of the binding site to HcPro and eIF4E, two different VPg partners [21], and also partially overlaps the TuMV VPg domain shown to be involved in eIF(iso)4E binding [17]. The tyrosine residue covalently linked to the viral RNA (position 60–64 depending on the virus) [56] is not located in a PDR.

### Disorder predictions in caliciviral VPgs

**Figure 5:**
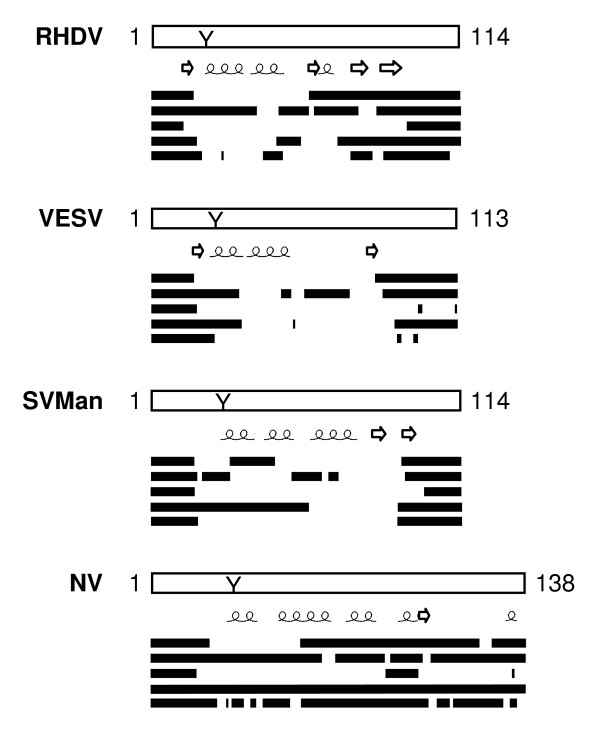
**Disorder predictions of caliciviral VPgs**. Five predictors were used: PONDR^® ^VLXT, FoldIndex^©^, DISOPRED2, VSL2, IUPred. The location of predicted disordered (in the order provided by the above-listed predictors) was schematically represented by lines along the VPg sequence. Numbering represents the VPg length. The consensus predicted α-helices and β-strands are indicated. The conserved tyrosine residue (Y) involved in VPg urydylylation is indicated. RHDV *Rabbit hemorrhabic disease virus *(*Lagovirus*), VESV *Vesicular exanthema of swine virus *(*Vesivirus*), SV Man *Sapporo virus Manchester virus *(*Sapovirus*) and NV *Norwalk virus *(*Norovirus*).

The *Caliciviridae *family comprises four genera of human and animal viruses [57] and possesses VPgs displaying intermediary lengths between those of sobemoviral and potyviral VPgs [23]. The VPg sequence of a member representative of each genus was analysed. NV VPg, which is the longest caliciviral VPg, was predicted to be fully disordered by most of the disorder predictors. For the three other caliciviral VPgs, most PDRs are conserved although the VPg sequence identities range from 25% to 36% (Figure 5). N-terminal extremities and C-terminal halves are always predicted to be disordered. In addition, several internal domains are also predicted to be disordered. The tyrosine residues involved in urydylylation (position 20–30 depending on the virus) [58] are generally not located in PDRs.

### α-MoRF predictions

Often, intrinsically disordered regions involved in protein-protein interactions and molecular recognition undergo disorder-to-order transitions upon binding [30-32,35,59-63]. A correlation has been established between the specific pattern in the PONDR^® ^VLXT curve and the ability of a given short disordered regions to undergo disorder-to-order transitions on binding [64]. Based on these specific features, an α-MoRF predictor was recently developed [60,65].

The application of the α-MoRF predictor to the set of 16 VPgs reveals that helix forming molecular recognition features are highly abundant in these proteins. Table 1 shows that there are 15 α-MoRFs in 12 VPgs. The regions of potyviral VPgs spanning residues 24–26 and 41–43 are always predicted to form α-MoRFs. By contrast, the putative α-MoRF regions are not conserved in sobemoviral and caliciviral VPgs, likely reflecting lower sequence conservation among these proteins but also suggesting diversity in the disordered state at intraspecies level. No α-MoRFs were predicted in VESV, RGMoV, SBMV and SCPMV VPgs. It should be pointed out, however, that not all MoRF regions share these same features and some of them may form β- or irregular structure rather than α-helices upon binding [61,62]. Therefore, predicted MoRFs only represent a fraction of the total numbers of potential MoRFs. According to secondary structure predictions, SBMV and SCPMV would form more preferentially β-MoRFs. In this respect, the prediction of α-MoRF in SeMV VPg, which is related to SBMV and SCPMV, was not expected.

**Table 1:** Location of predicted α-MoRFs in VPgs

Viral genus/family	Viral species	α-MoRFs
*Sobemovirus*	RYMV	14–31 56–73
	CoMV	1–18
	SeMV	43–60

*Potyvirus*	LMV	25–42
	PVY	25–42
	PVA	24–41 167–184
	TEV	26–43
	TuMV	25–42
	BYMV	26–43

*Caliciviridae*	RHDV	68–85
	SV Man	14–31
	NV	30–47 115–132

### CDF and CH-plot analyses

**Figure 6:**
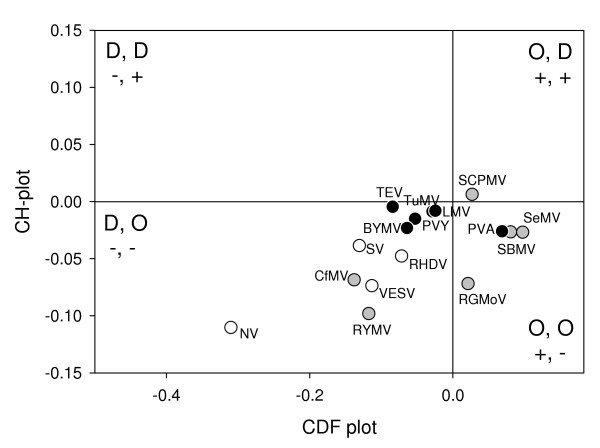
**Comparison of the PONDR^® ^CDF and CH-plot analyses of whole protein order-disorder *via *distributions of VPgs within the CH-CDF phase space**. Each spot represents a single VPg whose coordinates were calculated as a distance of this protein from the boundary in the corresponding CH-plot (Y-coordinate) and an average distance of the corresponding CDF curve from the boundary (X-coordinate). The four quadrants in the plot correspond to the following predictions: (-, -) proteins predicted to be disordered by CDF, but compact by CH-plot; (-, +) proteins predicted to be disordered by both methods; (+, -) contains ordered proteins; (+, +) includes proteins predicted to be disordered by CH-plot, but ordered by the CDF analysis. Open circles correspond to caliciviral VPgs, gray circles represent sobemoviral VPgs, whereas black circles correspond to potyviral VPgs.

In order to compare the disordered state of VPgs from the various viral genera, VPg sequences were analyzed by two binary predictors of intrinsic disorder, charge-hydropathy plot (CH-plot) [31,60] and cumulative distribution function analysis (CDF) [60]. These predictors classify entire proteins as ordered or disordered, as opposed to the previously described disorder predictors, which output disorder propensity for each position in the protein sequence. The usefulness of the joint application of these two binary classifiers is based on their methodological differences [60,66]. In Figure 6, each spot corresponds to a single protein and its coordinates are calculated as a distance of this protein from the folded/unfolded decision boundary in the corresponding CH-plot (Y-coordinate) and an average distance of the corresponding CDF curve from the order/disorder decision boundary (X-coordinate). Figure 6 shows that the majority of VPgs are predicted to be disordered: 11 VPgs including RYMV and LMV VPgs are located within the (-, -) quadrant suggesting that they belong to the class of native molten globules. Figure 6 shows that all *Caliciviridae *VPgs are predicted to be native molten globules, whereas VPgs from *Sobemoviruses *and *Potyviruses *are spread between different quadrants. Notably, PVA and SeMV VPgs are located in the (+,-) quadrant of the ordered proteins indicating that these binary methods failed to detect the experimentally demonstrated disorder of these two VPgs.

## Discussion

In this paper, we provide experimental evidences that RYMV and LMV VPgs contain intrinsically disordered regions. These findings, together with the previous reports documenting the disordered state of SeMV, PVY and PVA VPgs [25-27], suggest that intrinsic disorder may be a common and distinctive feature of sobemo- and potyviral VPgs. By carrying out an in-depth *in silico *analysis, we show that the disordered state of VPgs depend on the viral genera. Sobemoviral SeMV and RYMV VPgs appeared highly disordered with (i) 30% and 50% increases of their molecular masses estimated from SDS-PAGE compared to expected masses, respectively, and (ii) far-UV CD spectra with large negative ellipticities near 200 nm and low ellipticities at 190 nm. By contrast, the increase of the apparent molecular masses of potyviral VPgs from SDS-PAGE are moderate (<5% for LMV, approx. 10% for PVY and PVA) and the trends of far-UV CD spectra indicate partial disorder better suggesting short disordered regions included in globally ordered VPgs.

The experimentally observed disorder is also pointed out by complementary *in silico *analyses. However, quantitative assessment of disorder prediction strengths and precise location of consensus disordered regions turned out to be hectic. While LMV, PVY and PVA VPgs showed longer disordered segments, SeMV VPg showed short disordered segments whereas experimental results were similar to RYMV VPg. Moreover, binary predictors which are intended to allow a comparison of relative disordered states failed to detect disorder in several VPgs, including those for which the disordered state has been shown experimentally such as SeMV and PVA. However, it is important to notice that these predictors are meant to predict disorder on an entire protein basis, and SeMV and PVA not only have substantial ordered regions, but their disordered regions are in general shorter than those of the other proteins studied. These features could have easily tipped the balance towards an "ordered protein" prediction. Otherwise, the use of complementary disorder predictors induces difficulties to precisely map consensus disordered regions in VPgs, but this is due mainly to the fact that different disorder predictors are built upon slightly different definitions of disorder [41]. This is what makes these predictions complementary of each other.

The presence of intrinsically disordered (ID) regions was detected by five per-residue disorder predictors in 10–26 kDa VPgs. At intra-specific level in sobemo- and in potyviruses, the presence of intrinsic disorder regions was conserved independently from sequence conservation. Therefore, we enlarged our analysis to other genera, namely caliciviral VPgs that had never been suggested before to be disordered, and small VPgs (2 to 3 kDa) from *Picornaviridae *and *Comoviridae *where ID was also predicted (data not shown). By contrast to several domains in capsid and polymerase viral proteins, the disorder propensity had not been described so far as a common property of VPgs [67]. The methodology used by Chen and colleagues is likely not adapted to the highly diverse set of VPg sequences because it includes a first step of conserved domain identification before performing the disorder predictions.

VPg ID was rather predicted in several small patches (<30 residues) than in few large domains, this trend is common in short protein sequences with binding sites. These characteristics of variable degree of disorder, together with the complementarities of disorder definitions described above, may explain why discrepancies in location of PDRs were frequently observed. Still, all proteins showed a high predicted disorder content (percentage of disordered residues), ranging in average from 44% for sobemoviral to 60% for caliciviral VPgs (PONDR^® ^VSL2 predictions). Part of the hydrophobic residues of VPgs would be involved in the formation of additional secondary structure elements. We performed *in silico *detection of α-helix-forming molecular recognition features (α-MoRF) which mediate the binding of initially disordered domains with interaction partners [60]. Some α-MoRF domains were detected in the N-terminal regions of VPgs which were not reported to be interacting domains. By contrast, the first half of the C-terminal domain of RYMV VPg and the central domain of LMV VPg previously predicted to form α-helices [21,24] were not identified as α-MoRFs. These domains were predicted both to be disordered and to form α-helices. The α-helical propensities of RYMV VPgs, as observed in the presence of TFE concentration as low as 5% (Figure 2), suggest that some disordered regions in the isolated proteins may undergo a disorder-to-order transition upon association with a partner protein. Noteworthy, the only VPg structures available to date (*Picornaviridae*) were obtained either in the presence of a stabilizing agent [49] or in association with the viral RNA-dependent RNA polymerase (3D) which probably stabilized the VPg folded state [50,51].

The property of proteins to be intrinsically disordered confers to them the ability to bind to many different partners. These characteristics likely explain why many proteins critical in interaction networks (hub proteins) are intrinsically disordered [36,45]. In RYMV VPg, the resistance-breaking positions 48 and 52 suggested to be involved in eIF(iso)4G interaction are located in a putative α-helix also predicted to be disordered. The same result is obtained with LMV VPg where resistance-breaking sites involved in eIF4E interaction are located in the central domain predicted to contain two α-helices and to display disorder features. Analysis of other potyviral VPgs suggests that domains associated with virulence are often disordered with some residual structure. Besides their interactions with eIF4Es, potyviral VPgs were found to interact with a variety of host factors such as poly(A)-binding protein [68,69], eIF4G [18] and eukaryotic elongation factor eEF1A [70]. Multiple *in vitro *interactions of VPgs with eIF4GI [71], eIF3 [72] and eIF4A [73], and others proteins belonging to the translation initiation complex, were also shown for *Caliciviridae *members. Potyviral VPgs were also reported to interact with several viral proteins such as NIb, HC-Pro, CI and CP [9,68,74].

As underlined in the introduction, VPgs are multifunctional proteins. At least part of their functions implies interactions with eIFs, with the VPg/eIF4E interaction having been shown to enhance the *in vitro *translation of viral RNA [22,75]. VPgs were suggested to mimic the mRNA 5'-linked cap recruiting the translation initiation complex. Besides, a ribonuclease activity of VPgs was reported. It might contribute to host RNA translation shutoff [76]. VPg-eIF interactions were also suggested to be involved in other key steps in the viral cycle [1]. In *Picornaviridae*, it was established that VPg is involved in genome replication, its uridyl-form acting as primer for complementary strand synthesis [77,78]. An additional role of potyviral VPg-eIF4E interactions in plant cell-to-cell movement *via *eIF4G and microtubules was also suggested [2,79]. VPg could participate to a putative vascular movement complex to cross the plasmodesmata and may facilitate virus unloading [9,80]. Thus, VPg might be involved in key steps of the viral cycle such as replication, translation and movement. Additionally, ID VPg was reported to be necessary to the processing of SeMV polyprotein by viral protease [25]. ID might explain how a unique protein can perform and regulate these different biological functions. PDRs might give to the VPg the necessary plasticity to fit surface overlaps with various partners.

## Conclusion

Experimentally, we showed that RYMV and LMV VPgs contain both intrinsically disordered domains but with different disordered states. Using *in silico *analyses, ID domains were predicted to occur in 14 VPgs of sobemo-, poty-and caliciviruses. Although highly diverse, VPgs share the common feature of possessing ID domains. These structural properties of VPgs are more conserved than what could be anticipated from their sequence homologies. However, comparative analyses at intra-and interspecies levels showed the diversity of intrinsic disorder in VPgs.

Like many IDPs, VPg ID domains may play a role in protein interaction networks, interacting in particular with translation initiation factor eIFs to perform key steps of the viral cycle (replication, translation and movement).

## Methods

### Purification of recombinant RYMV and LMV VPgs

The VPg-encoding region in the RYMV ORF2a was amplified by PCR from FL5 infectious clone [81] by using the primers FCIaVPgH 5'ATATCCATGGGATCCCA TTTGAGATTTACGGC (containing a *Nco*I site and RYMV nucleotides 1587–1607) and RCIaVPgH 5'TGCAAGATCTCTCGATATCAACATCCTCGCC (containing a *Bgl*II site and sequence complementary to RYMV nucleotides 1823–1803). The resulting fragment was cloned into the *Nco*I and *Bgl*II sites of pQE60 as a 6-His C-terminal fusion (Qiagen) and the construct was sequenced. The resulting expression plasmid was used to transform the *E. coli *strain M15-pRep4 (Qiagen). After induction with 0.5 mM isopropyl-1-thio-β-D-galactopyranoside at 25°C for 5 h, the cells from 1 L culture in LB medium were harvested by centrifugation and frozen at -80°C. Cells were thawn, resuspended in 30 mL of purification buffer (50 mM Tris-HCl, pH 8.0, 300 mM NaCl, 10% glycerol), disrupted with a French press (Thermo) and centrifuged at 18000 rpm for 30 min. The supernatant was filtered (0.5 μm filters) and purification of the VPg in native conditions was carried out using a nickel-loaded HiTrap IMAC HP column (GE Healthcare) followed by gel filtration step onto a HR10/30 Superdex 75 column (GE Healthcare) in 50 mM Tris-HCl, pH 8.0, 300 mM NaCl, 5% glycerol.

LMV VPg was produced in *E. coli *using the pTrcHis plasmid as expression vector as already described [18]. The N-terminal His-tagged protein was found to be expressed in the soluble fraction of the bacterial lysate and was purified as described above, except that 50 mM Tris-HCl pH 8, 800 mM NaCl, 10% glycerol, 2 mM β-mercaptoethanol was used as the affinity chromatography buffer, and 20 mM Tris-HCl pH 8, 800 mM NaCl, 5% glycerol as gel filtration buffer.

### Circular dichroism analyses

Freshly purified protein samples were used for CD analyses. Sample buffer was changed by eluting the protein from a PD10 desalting column (GE Healthcare) using 10 mM sodium phosphate buffer (pH 8.0), supplemented with 300 mM or 500 mM NaF for RYMV or LMV VPgs respectively. After centrifugation, the protein concentration was determined using a ND-1000 Spectrophotometer (NanoDrop Technologies) and an extinction coefficient of 7,780 and 18,490 M^-1^cm^-1 ^for RYMV and LMV VPgs respectively. Far UV-CD spectra were recorded with a chirascan dichrograph (Applied Photophysics) in a thermostated (20°C) quartz circular cell with a 0.5 mm path length, in steps of 0.5 nm. All protein spectra were corrected by subtraction of the respective buffer spectra. The mean molar ellipticity values per residue were calculated using the manufacturer software. Structural variations of the native protein samples were monitored by recording successive CD spectra after addition of 2,2,2-trifluoroethanol (TFE, Sigma) in the 5–30% range (vol:vol).

### VPg sequences

Sequences for this study were obtained from the viral genome resources at NCBI . Sequence accession numbers are: *Sobemovirus *(RYMV AJ608219, CoMV NC_002618, RGMoV NP_736586, SBMV NP_736583, SCPMV NP_736598, SeMV NP_736592), *Potyvirus *(LMV NP_734159, PVY NP_734252, PVA NC_004039, TEV NP_734204, TuMV NC_002509, BYMV NC_003492), and *Caliciviridae *(RHDV NP_740330, VESV NP_786894, SV Man X86560, NV NP_786948).

### Disorder predictions

Seven programs were used to predict the disorder tendency of VPgs. PONDR^®^, Predictors of Natural Disordered Regions, version VLXT is a neural network principally based on local amino acid composition, flexibility and hydropathy [82]. FoldIndex^© ^is based on charge and hydropathy analyzed locally using a sliding window [83]. DISOPRED2 is also a neural network, but incorporates information from multiple sequence alignments generated by PSI-BLAST [44]. PONDR^® ^VSL2 has achieved higher accuracy and improved performance on short disordered regions, while maintaining high performance on long disordered regions [84]. IUPred uses a novel algorithm that evaluates the energy resulting from inter-residue interactions [85]. PONDR^® ^VLXT and VSL2 as well as DISOPRED2 were all trained on datasets of disordered proteins, while FoldIndex^© ^and IUPred were not. Binary classifications of VPgs as ordered or disordered were performed using CDF and CH-plot analyses. Cumulative distribution function curves or CDF curves were generated for each dataset using PONDR^® ^VLXT scores for each of the VPgs [60]. Charge-hydropathy distributions (CH-plots) were also analyzed using the method described in Uversky et al. [31].

### α-MoRF predictions

The predictor of α-helix forming Molecular Recognition Features, α-MoRF, focuses on short binding regions within regions of disorder that are likely to form helical structure upon binding [60,65]. It utilizes a stacked architecture, where PONDR^® ^VLXT is used to identify short predictions of order within long predictions of disorder and then a second level predictor determines whether the order prediction is likely to be a binding site based on attributes of both the predicted ordered region and the predicted surrounding disordered region. An α-MoRF prediction indicates the presence of a relatively short (20 residues), loosely structured helical region within a largely disordered sequence [60,65]. Such regions gain stable structure upon a disorder-to-order transition induced by binding to partner.

## Competing interests

The authors declare that they have no competing interests.

## Authors' contributions

EH carried out experiments and drafted the manuscript. YB participated in the design, performed protein purifications and far UV-CD analyses. TM and JW participated in LMV VPg analyses. SL participated in predictive analyses. VNU performed CDF and CH-plot analyses. FD and AVD performed the mass spectrometry analyses. PR performed α-MoRF analyses. ND and DF participated in the study design and coordination and helped to draft the manuscript. All authors read and approved the final manuscript.
